# Supplementary material for: Stabilizing ultrasmall Au clusters for enhanced photoredox catalysis
Source: Nat Commun. 2018 Apr 18;9:1543. doi: 10.1038/s41467-018-04020-2 (PMC5906565; doi:10.1038/s41467-018-04020-2)
Supplement: Supplementary file 1 — Supplementary Information [file 41467_2018_4020_MOESM1_ESM.pdf]

Supplementary Information

Title: Stabilizing ultrasmall Au clusters for enhanced photoredox catalysis

Authors: Weng *et al.*

Supplementary Methods

(a) Synthesis of $Au_{25}(SG)_{18}$ clusters. A mixture of glutathione-protected Au clusters was synthesized according to the literaturewith some modifications. Briefly, glutathione (reduced form, 1 mmol) was added to methanol (50 mL) containing $HAuCl_4{\cdot}3H_2O$ (0.25 mmol). Under vigorous stirring, an ice-cold $NaBH_4$ aqueous solution (0.2 M, 12.5 mL) was added and aged for 1 h. The obtained precipitate was thoroughly washed with methanol and dried in vacuum at room temperature to obtain a mixture of gold clusters. The mixture (4.9 mg) was dissolved in an aqueous solution (7 mL) containing glutathione (130.7 mg) and stirred at 328 K under air bubbling for 6-9 h to obtain $Au_{25}(SG)_{18}$ clusters. To remove excess glutathione, the obtained solution containing the $Au_{25}(SG)_{18}$ clusters was loaded into a dialysis membrane (MWCO 8000) and stirred slowly at < 283 K for 12 h. The precipitate formed during dialysis was removed with a filter (pore size, 0.2 μm). It was confirmed by polyacrylamide gel electrophoresis that the obtained solution contains no other clusters. (b) Synthesis of SiO_2-$Au_{25}(SG)_{18}$ clusters-BPEI composite (SASB): 0.1 g positively charged BPEI modified-SiO_2 spheres were dispersed in 50 mL DI water by ultrasonication. Then, 5 mL negative charged $Au_{25}(SG)_{18}$ clusters (0.2 mg mL^{-1}) were added dropwise to this dispersion. SAB were isolated by centrifugation, washed with ethanol and DI water repeatedly, and finally dried in air. (c) Synthesis of BPEI modified-metal oxdie nanoparticles (ZnO and ZrO_2): 0.4 g metal oxdie nanoparticles, such as ZnO and ZrO_2, were dispersed in 200 mL ethanol by ultrasonication with adding 4 mL BPEI solution (86 mg mL^{-1}). The mixtures were heated at 333 K in an oil bath with constant stirring for 4 h and cooled to room temperature. The BPEI modified-metal oxdie nanoparticles were isolated by centrifugation, washed with ethanol and DI water repeatedly, and finally dried in air. (d) Synthesis of metal oxdie nanoparticles (ZnO and ZrO_2)-Au GSH clusters-BPEI composites (MABs): 0.1 g positively charged BPEI modified-metal oxdie nanoparticles (ZnO and ZrO_2) were dispersed in 50 mL DI water by ultrasonication. Then, 5 mL negative charged Au GSH clusters (0.2 mg mL^{-1}) were added dropwise to this dispersion. SAB were isolated by centrifugation, washed with ethanol and DI water repeatedly, and finally dried in air. (e) Synthesis of SiO_2-Au GSH clusters-pH@TiO_2 nanostructures by hydrolysis of TIP: 0.08 g of as-prepared SAP composites were firstly dispersed in 9.74 mL of ethanol by ultrasonication. Then, 0.08 g of HDA (90%) and 0.2 mL of ammonia were added into the dispersion under stirring at room temperature. After 1 min, different amounts of TIP (97 %) were added to the dispersion under stirring. After reaction for 10 min, the core-shell SiO_2-Au GSH clusters-pH@TiO_2 composites were collected by centrifugation and then washed several times with DI water and ethanol.

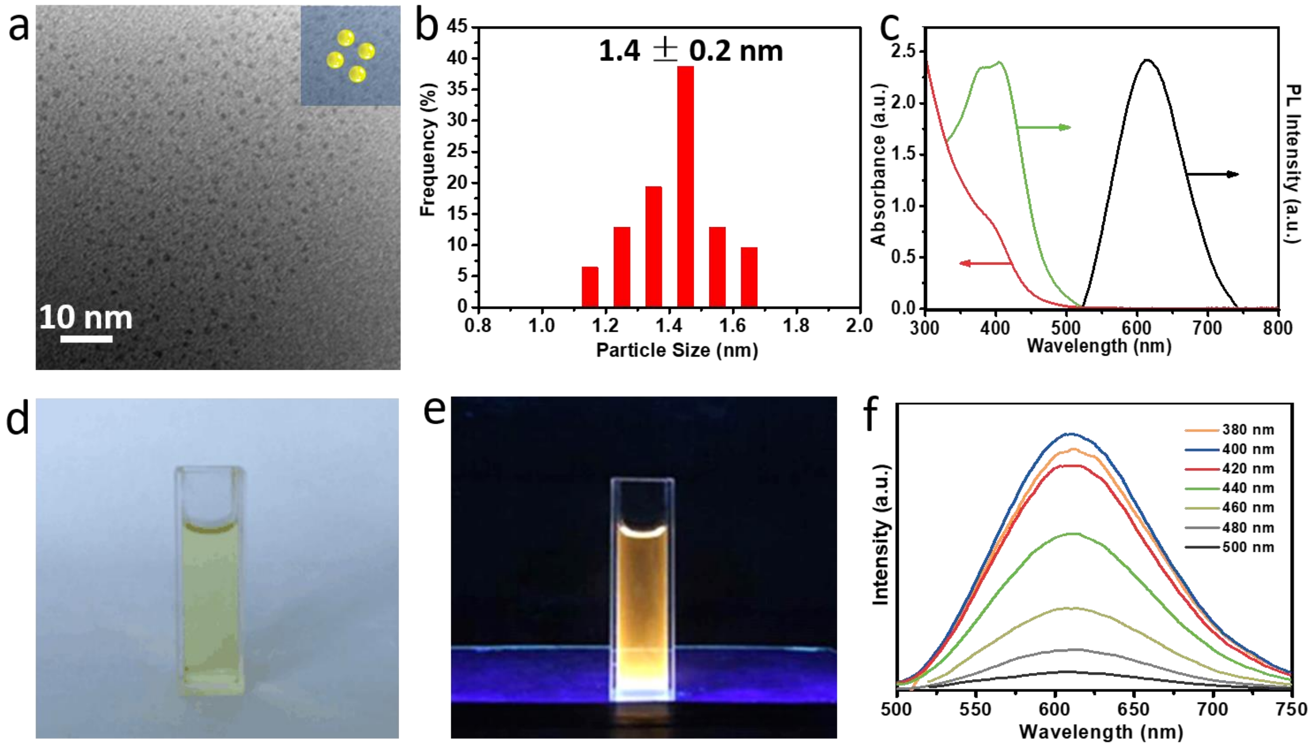

Supplementary Figure 1. Structural characterizations of Au GSH clusters (a) TEM image and (b) size distribution histogram of Au GSH clusters; (c) UV-vis absorption (red), excitation spectrum (green) and emission spectrum (black) of Au GSH clusters in aqueous suspension; digital photographs of Au GSH clusters aqueous solution under the (d) daylight and (e) blacklight illumination; (f) emission spectra of Au GSH clusters aqueous solution under different excitation wavelengths from 380 nm to 500 nm. The insets of a are the model illustrations of Au GSH clusters.

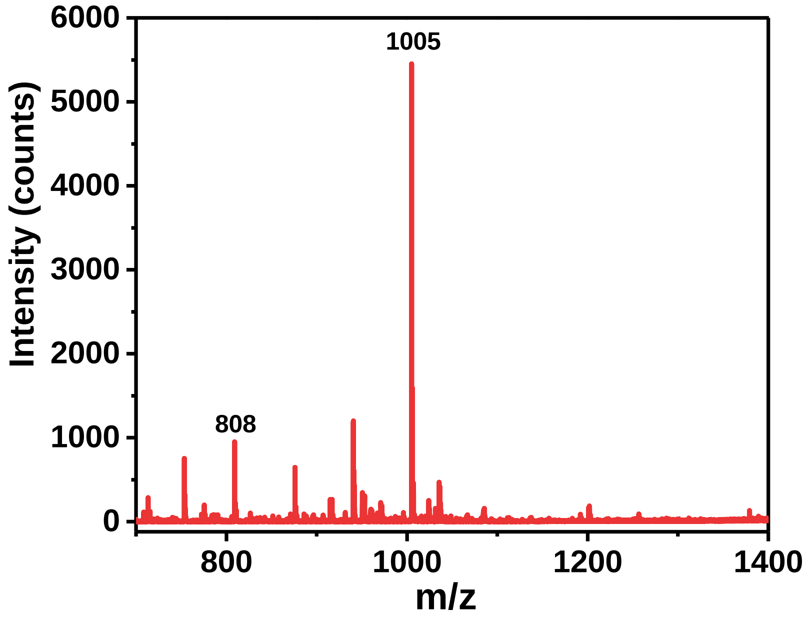

Supplementary Figure 2. ESI mass spectrum of Au GSH clusters, which gives characteristic peaks at m/z 808 and 1005 due to the presence of fragments of $[Au(SG)_2\text{-}H]^{-1}$ and $[Au_2(SG)_2\text{-}H]^{-1}$, respectively.

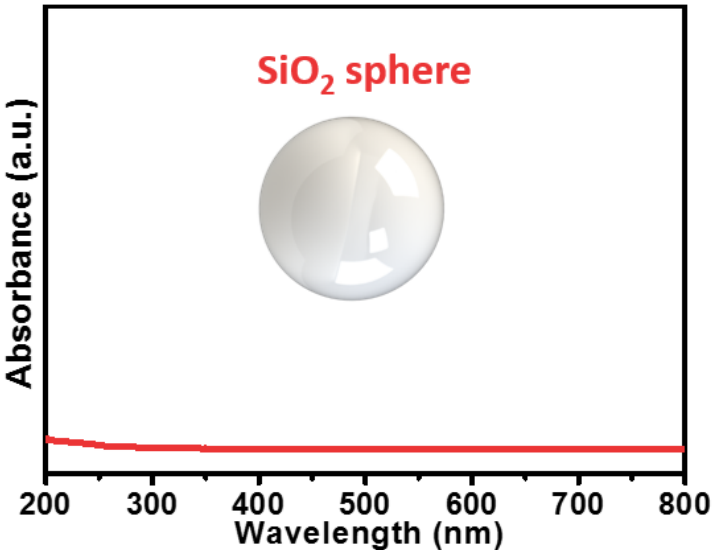

Supplementary Figure 3. UV-vis diffuse reflectance spectrum (DRS) of blank SiO_2 spheres; the inset is the corresponding model illustration.

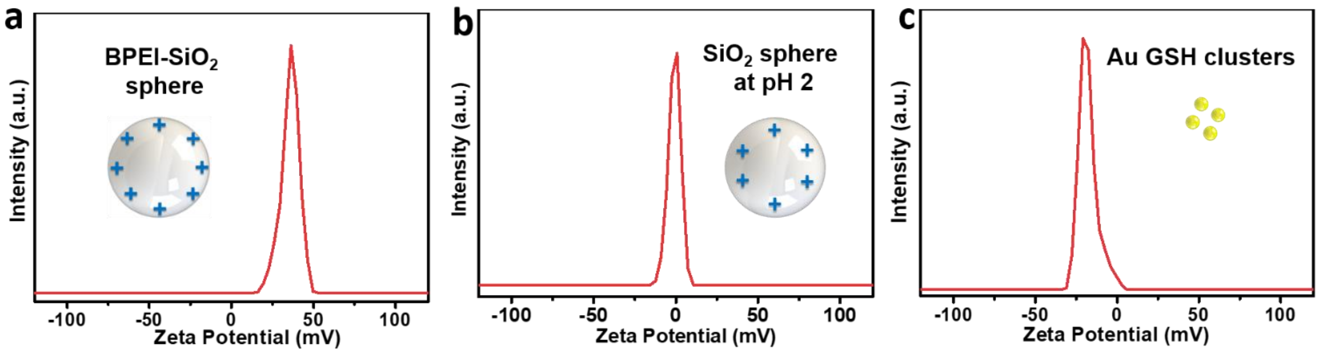

Supplementary Figure 4. Zeta potential measurement of different samples The zeta potential (ξ) values of (a) BPEI-SiO_2 spheres; (b) SiO_2 sphere at pH 2 and (c) Au GSH clusters in water. The insets of a-c are the corresponding model illustrations.

Note: The ξ value of SiO_2 spheres at pH 2 is +5 mV. The ξ values of BPEI-SiO_2 spheres and Au GSH clusters in water without adjusting pH values are demonstrated to be +36 mV and –21 mV, respesctively.

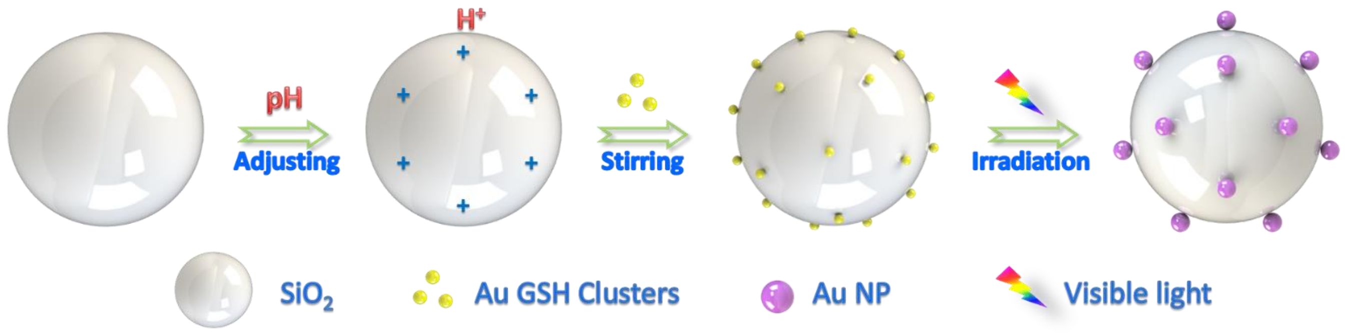

Supplementary Figure 5. Schematic illustration of synthesis procedure for SiO_2-Au GSH clusters composites (SAP) by pH adjusting process and the photostability testing of as-prepared SAP.

Supplementary Table 1. The loading amounts of Au GSH clusters in SAB and SAP samples quantified by ICP-OES.

Samples	SAB	SAP
Loading amount of Au GSH clusters	0.86%	0.28%

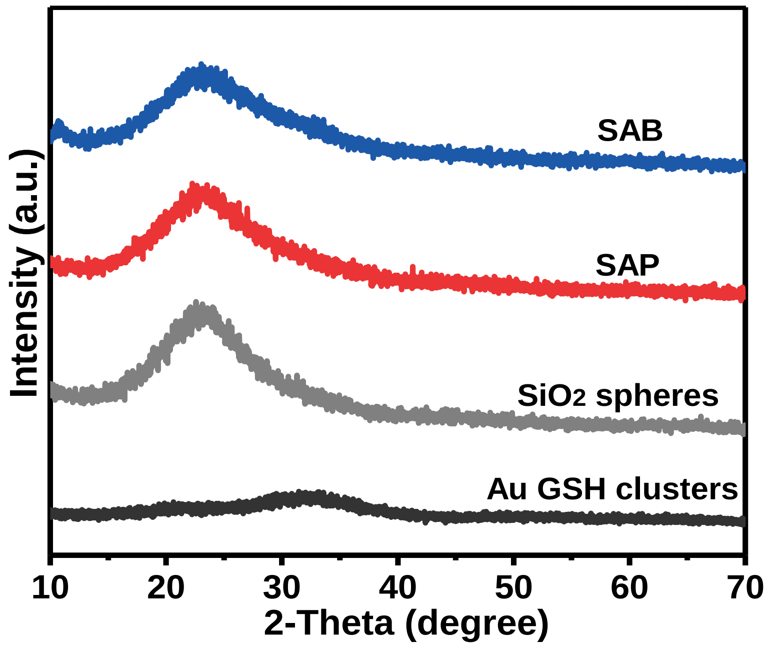

Supplementary Figure 6. Crystal structures of different samples XRD patterns of Au GSH clusters, SiO_2 spheres, SiO_2-Au GSH clusters-BPEI composites (SAB) and SAP.

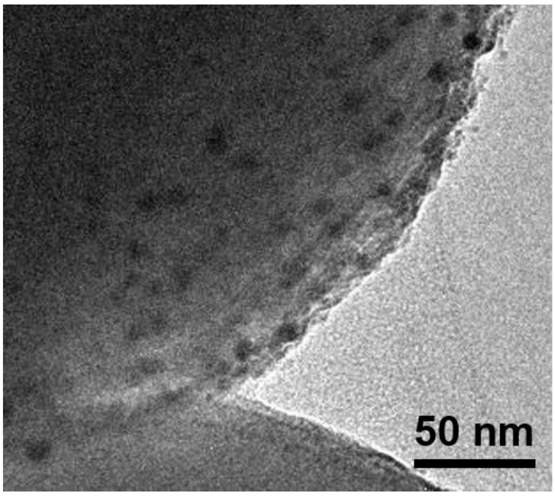

Supplementary Figure 7. HRTEM image of SAP after 10 h visible light irradiation (λ > 420 nm).

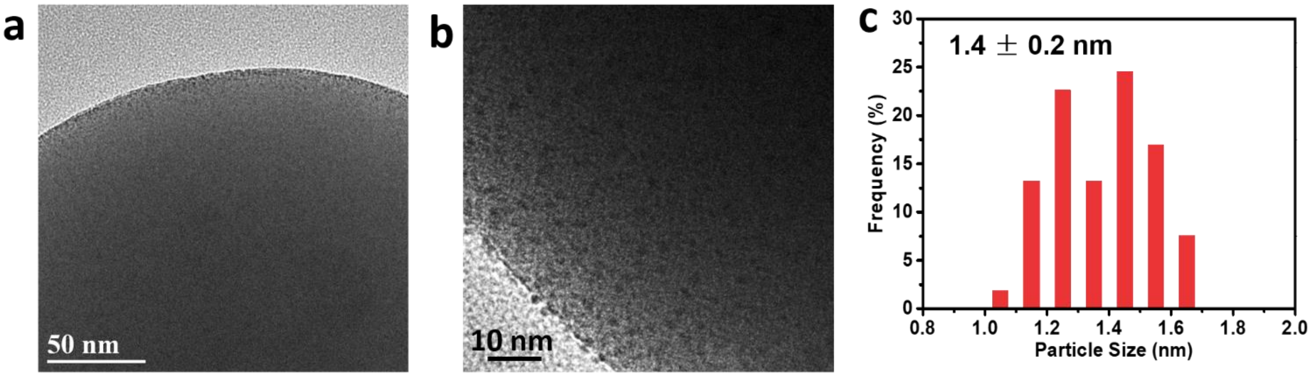

Supplementary Figure 8. Morphology and size information of Au GSH clusters (a) TEM image and (b) HRTEM image of SiO_2-Au GSH clusters-BPEI composites (SAB) after visible light irradiation (λ > 420 nm) for 24 h; (c) size distribution histogram of Au GSH clusters over SAB after visible light irradiation (λ > 420 nm) for 24 h.

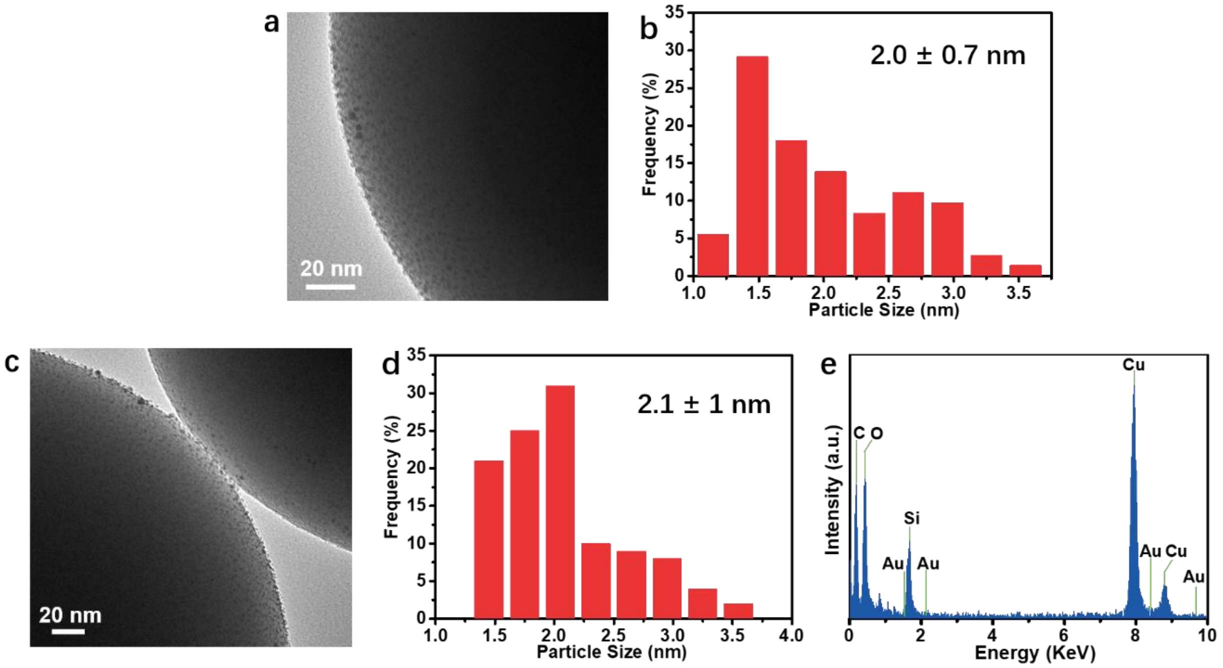

Supplementary Figure 9. Characterizations of Au GSH clusters in different samples HRTEM images of SAB after visible light irradiation (λ > 420 nm) for (a) 36 h and (c) 48 h; size distribution histograms of Au GSH clusters over SAB after visible light irradiation (λ > 420 nm) for (b) 36 h and (d) 48 h; (e) EDX spectrum of SAB originated from Supplementary Fig. 9c.

Note: The EDX spectrum in Supplementary Fig. 9e evidences the presence of Au, O and Si elements over SAB sample and the detected element Cu can be attributed to the use of Cu grid, which serves as the support for TEM analysis.

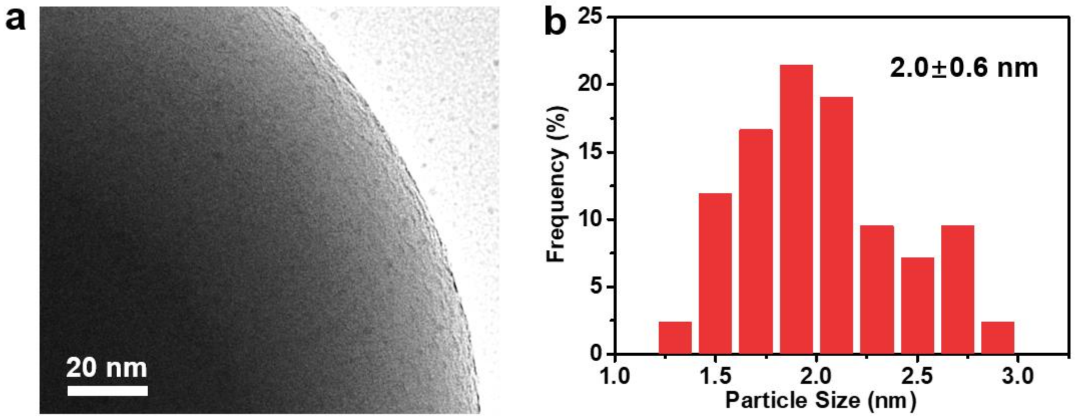

Supplementary Figure 10. Morphology and size information of Au GSH clusters (a) HRTEM image and (b) size distribution histogram of Au GSH clusters over BPEI modified SAP composite after visible light irradiation (λ > 420 nm) for 10 h.

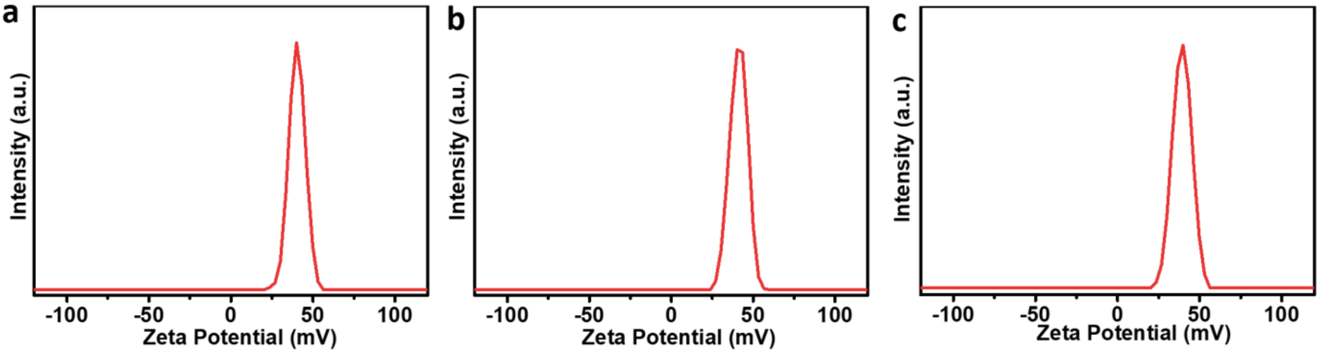

Supplementary Figure 11. Zeta potential measurement of different samples The zeta potential (ξ) values of (a) ZnO-BPEI; (b) ZrO_2-BPEI and (c) rutile TiO_2-BPEI in water without adjusting pH values.

Note: After the modification of BPEI, all of the different metal oxides are positively charged and the ξ values of ZnO-BPEI, ZrO_2-BPEI and rutile TiO_2-BPEI are demonstrated to be +40 mV, +41 mV and +39 mV, respectively.

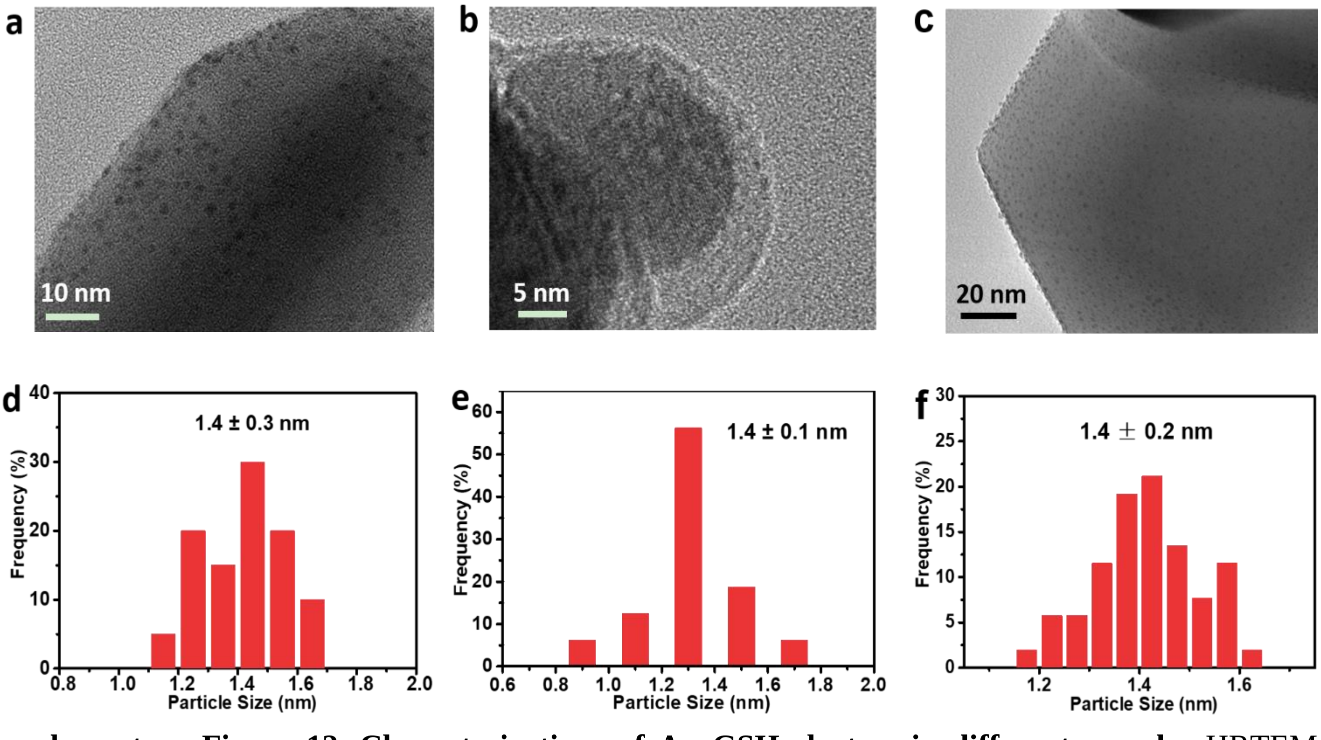

Supplementary Figure 12. Characterizations of Au GSH clusters in different samples HRTEM images of (a) ZnO-Au GSH clusters-BPEI composites, (b) ZrO_2-Au GSH clusters-BPEI composites and (c) rutile TiO_2-Au GSH clusters-BPEI composites after visible light irradiation ($\lambda > 420$ nm) for 10 h; size distribution histograms of Au GSH clusters over (d) ZnO-Au GSH clusters-BPEI composites, (e) ZrO_2-Au GSH clusters-BPEI composites and (f) rutile TiO_2-Au GSH clusters-BPEI composites after visible light irradiation ($\lambda > 420$ nm) for 10 h.

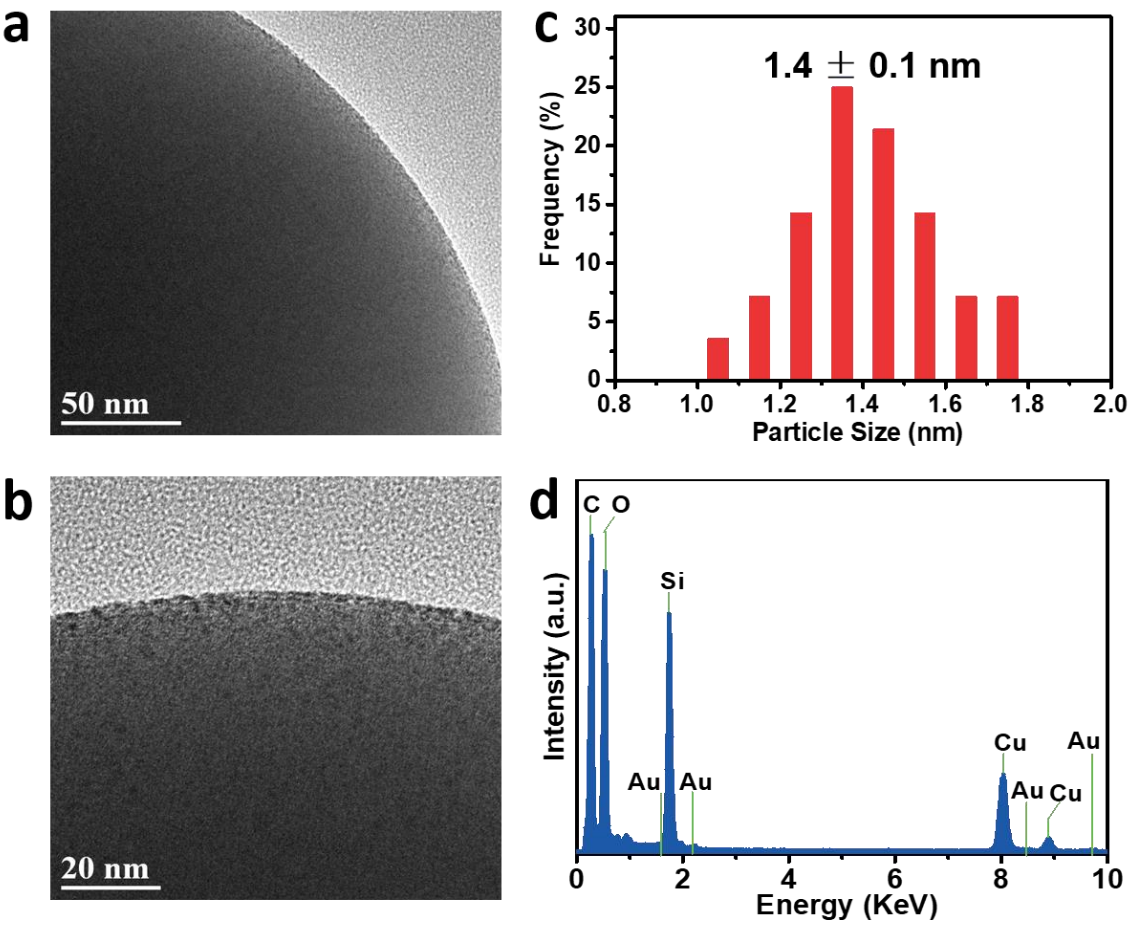

Supplementary Figure 13. Characterizations of Au GSH clusters in SASB sample (a) TEM image and (b) HRTEM image of SiO_2-$Au_{25}(SG)_{18}$ clusters-BPEI composites (SASB) after visible light irradiation (λ > 420 nm) for 10 h; (c) size distribution histogram of Au GSH clusters over SASB after visible light irradiation (λ > 420 nm) for 10 h; (d) the EDX spectrum of SASB originated from Supplementary Fig. 13b.

Note: The EDX spectrum in Supplementary Fig. 13d evidences the presence of Au, O and Si elements over SAB sample and the detected element Cu can be attributed to the use of Cu grid, which serves as the support for TEM analysis.

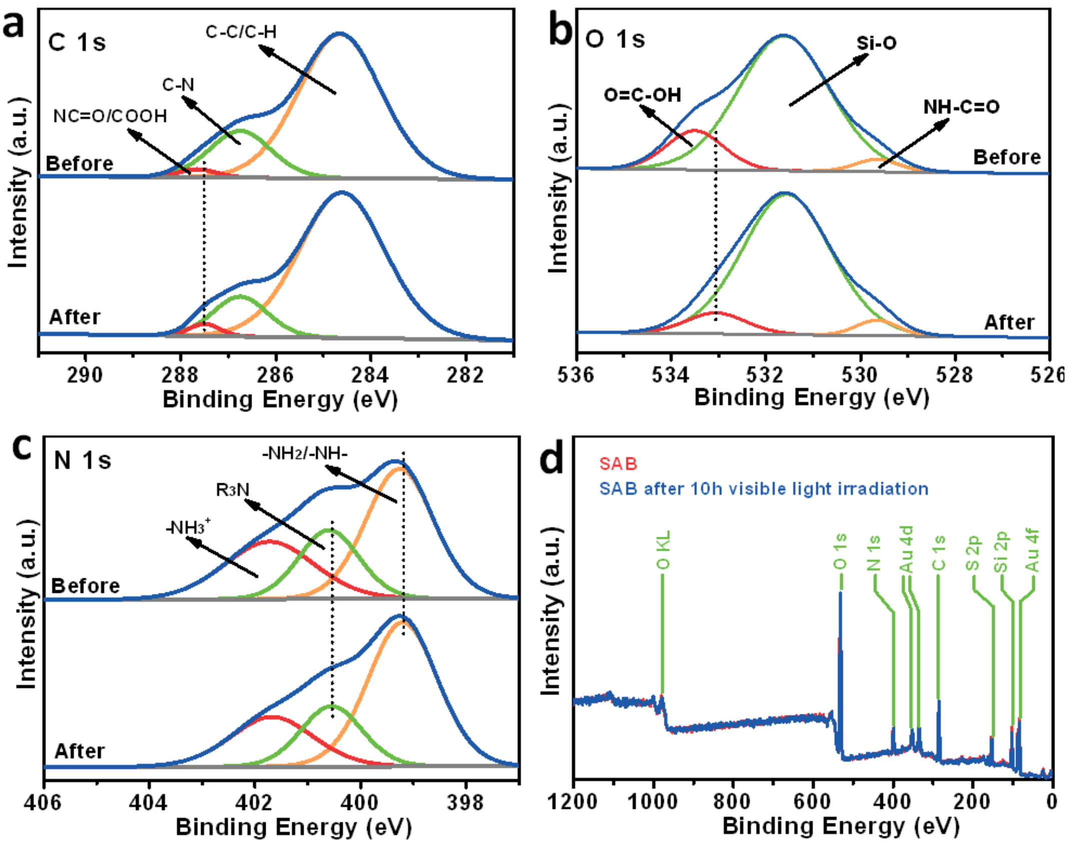

Supplementary Figure 14. High-resolution XPS spectra of (a) C 1s, (b) O 1s, (c) N 1s and (d) survey XPS spectra of SAB composites before/after 10 h visible light irradiation ($\lambda > 420$ nm).

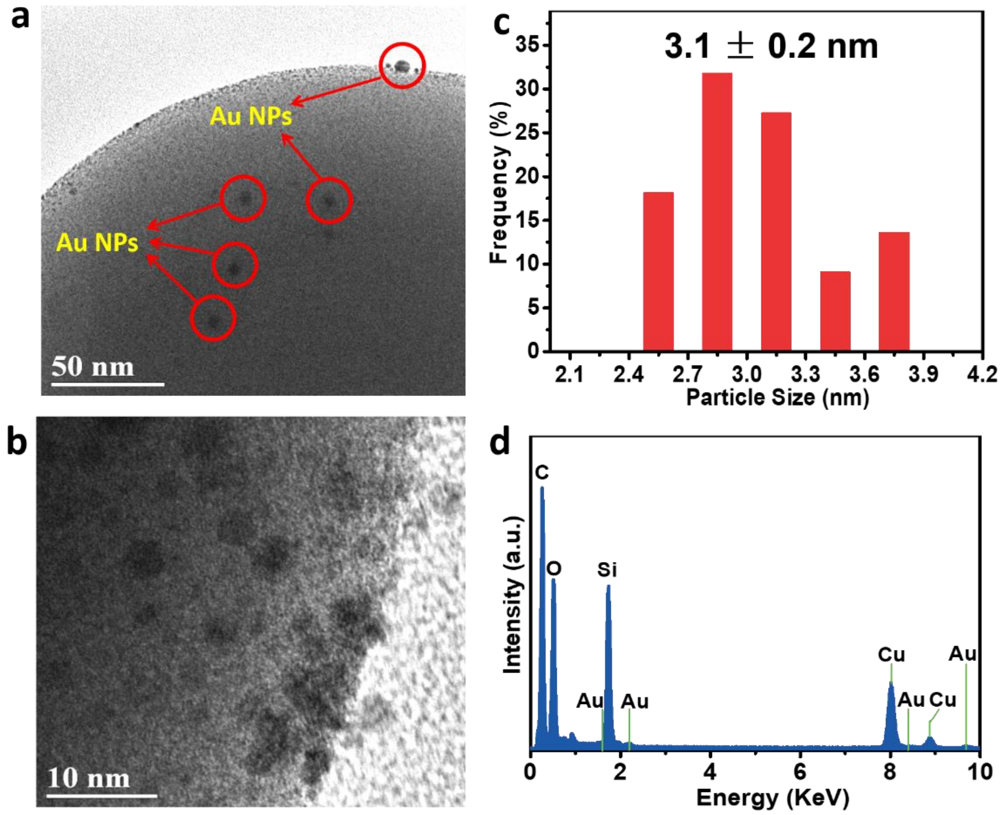

Supplementary Figure 15. Characterizations of Au GSH clusters in SAA composites (a) TEM image and (b) HRTEM image of SiO_2-Au GSH clusters-APTES composites (SAA) after visible light irradiation (λ > 420 nm) for 10 h; (c) size distribution histogram of Au GSH clusters over SAA after visible light irradiation (λ > 420 nm) for 10 h; (d) the EDX spectrum of SAA originated from Supplementary Fig. 15b.

Note: The EDX spectrum of SAA in Supplementary Fig. 15d suggests that the presence of Au, O and Si elements and the detected element Cu can be attributed to the use of Cu grid, which serves as the support for TEM analysis.

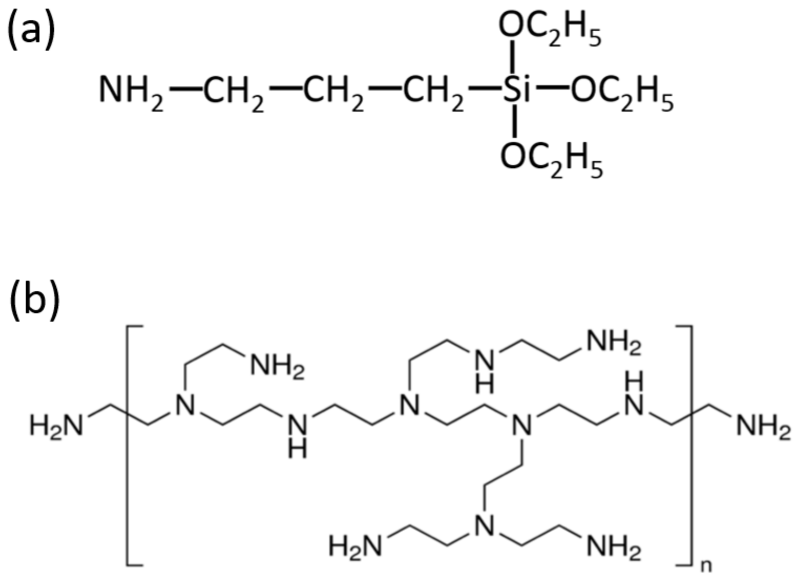

Supplementary Figure 16. The structural formulas of (a) APTES and (b) BPEI.

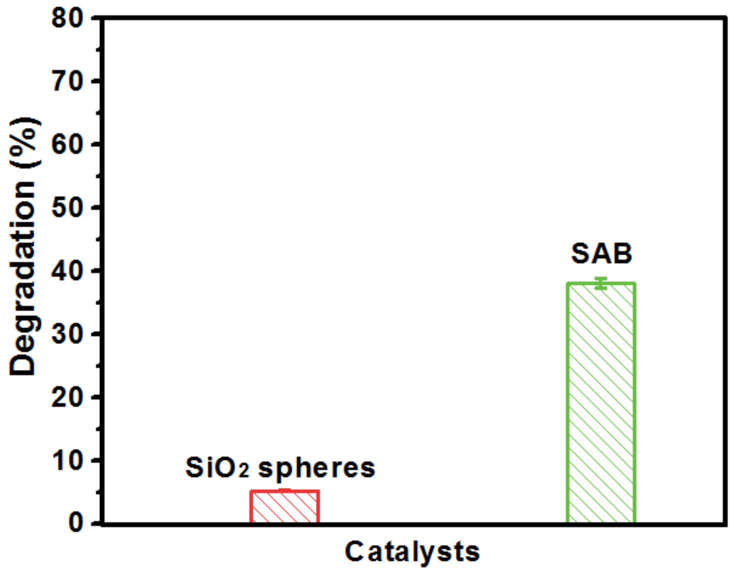

Supplementary Figure 17. Photocatalytic performances of SiO_2 spheres and SAB Photodegradation of RhB over bare SiO_2 spheres and SAB composites under visible light irradiation ($\lambda > 420$ nm) for 10 h. Note that the error bars represent the photoactivity s.d. values calculated from triplicate experiments.

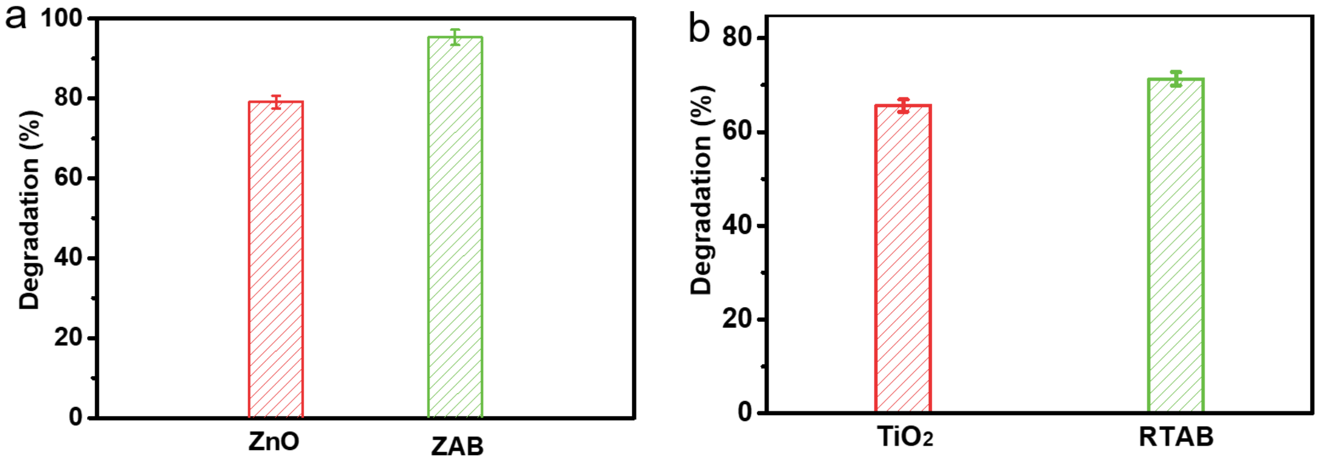

Supplementary Figure 18. Photocatalytic performances of various samples Photodegradation of RhB over (a) bare ZnO and ZnO-Au GSH clusters-BPEI composites (ZAB) for 9 h and (b) rutile TiO_2 and rutile TiO_2-Au GSH clusters-BPEI composites (RTAB) for 6 h under visible light irradiation ($\lambda >$ 420 nm). Note that the error bars represent the photoactivity s.d. values calculated from triplicate experiments.

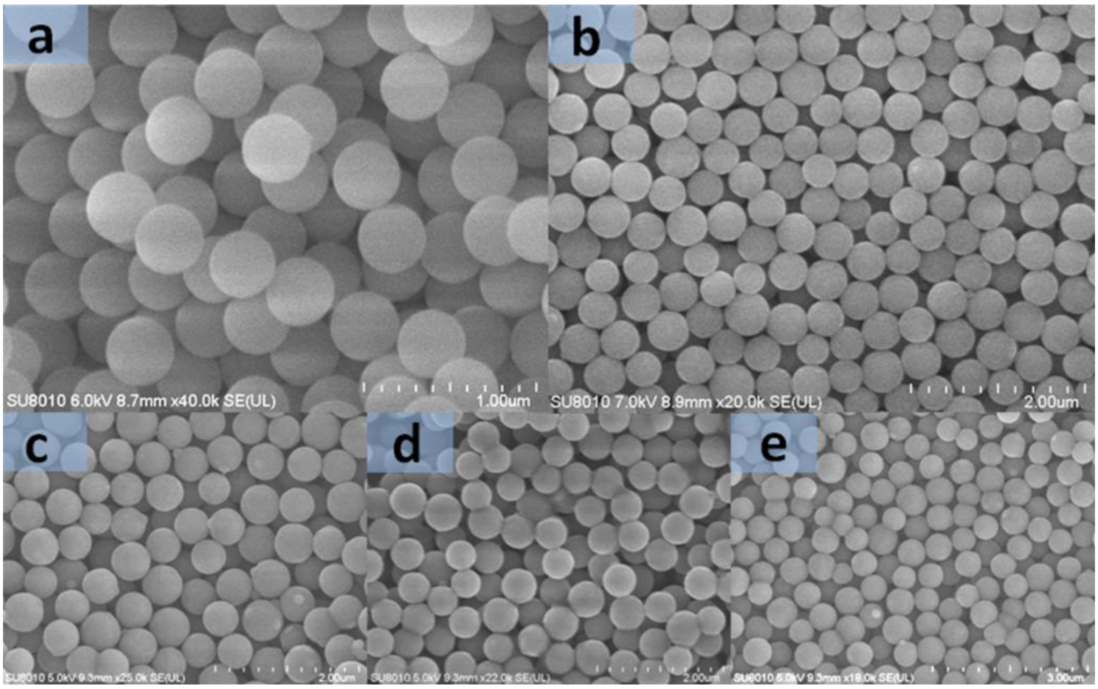

Supplementary Figure 19. Morphology information of different samples SEM images of (a) bare SiO_2 spheres and SiO_2-Au GSH clusters-BPEI@TiO_2 composites (SABT) with different TiO_2 shell thickness: (b) SABT-0.05, (c) SABT-0.1, (d) SABT-0.15 and (e) SABT-0.2.

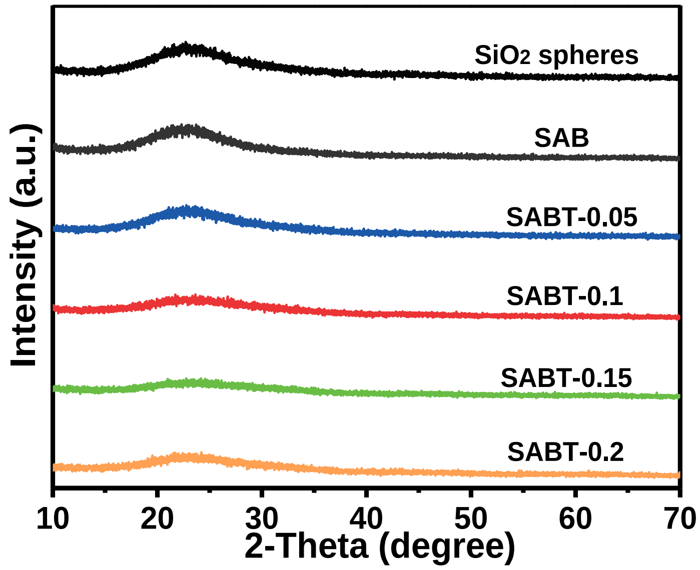

Supplementary Figure 20. XRD patterns of SiO_2 spheres, SAB and SABT composites with different TiO_2 shell thickness.

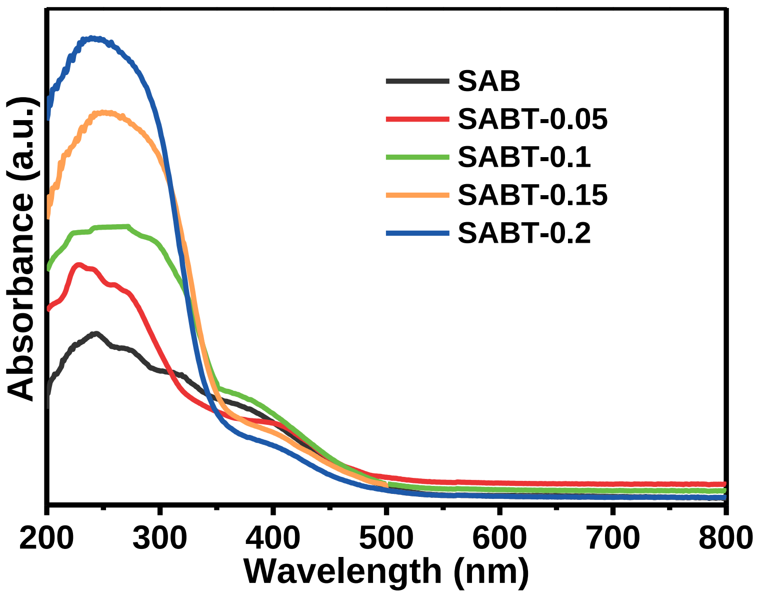

Supplementary Figure 21. DRS spectra of SAB and SABT composites with different TiO_2 shell thickness.

Supplementary Table 2. **The reaction rate constants** for photocatalytic degradation of RhB over different samples.

Samples	SiO_2	SAB	SABT-0.05	SABT-0.1	SABT-0.15	SABT-0.2	TAB	TiO_2
Reaction rate constants (min^{-1})	0	0.0143	0.0375	0.0646	0.119	0.0537	0.00645	0.000274

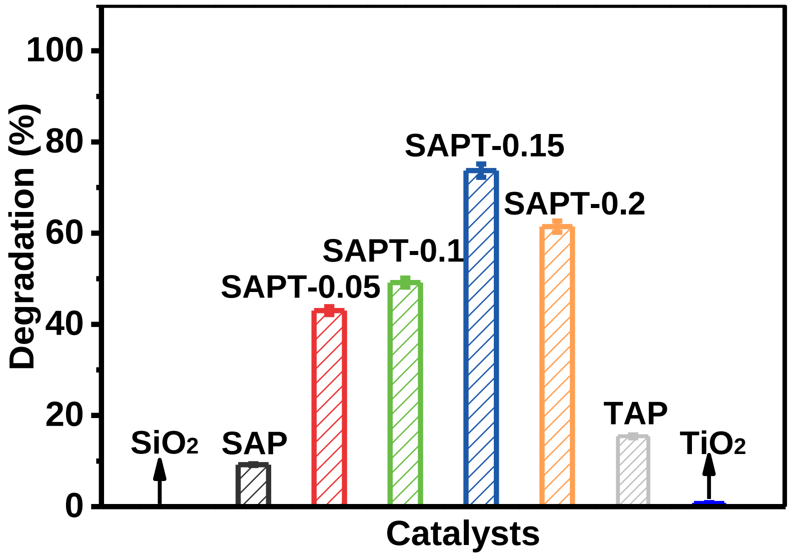

Supplementary Figure 22. Normalized photocatalytic performances of various samples Normalized photocatalytic degradation of RhB over blank SiO_2 spheres, SAP, TAP and SAPT composites with different TiO_2 shell thickness under visible light irradiation (λ > 420 nm) for 0.5 h. Note that the error bars represent the photoactivity s.d. values calculated from triplicate experiments.

Note: Due to the addition amount of Au GSH clusters in SAP is different from that of SAB (Supplementary Table 1), the photocatalytic activity of RhB degradation over SAP, TAP and SAPT composites with different TiO_2 shell thickness has been normalized with respect to the loading amount of Au GSH clusters in SAB for a fair comparison.

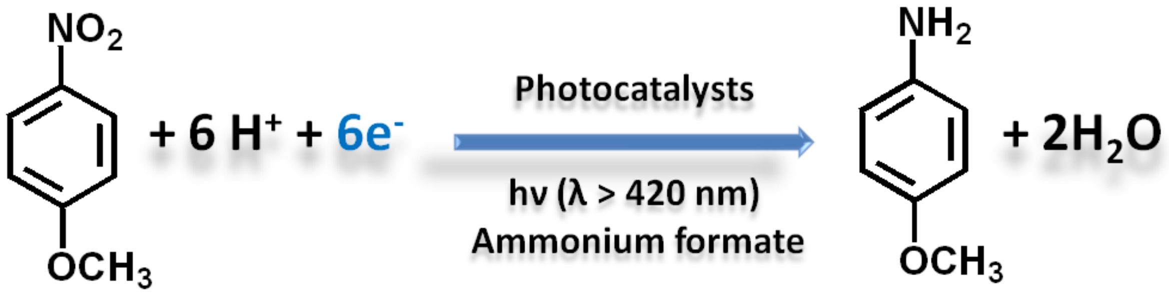

Supplementary Figure 23. The formula for photocatalytic reduction of aromatic nitro compound *p*-methoxy nitrobenzene to corresponding amino compound *p*-methoxy aniline over the SABT composites under visible light illumination (λ > 420 nm).

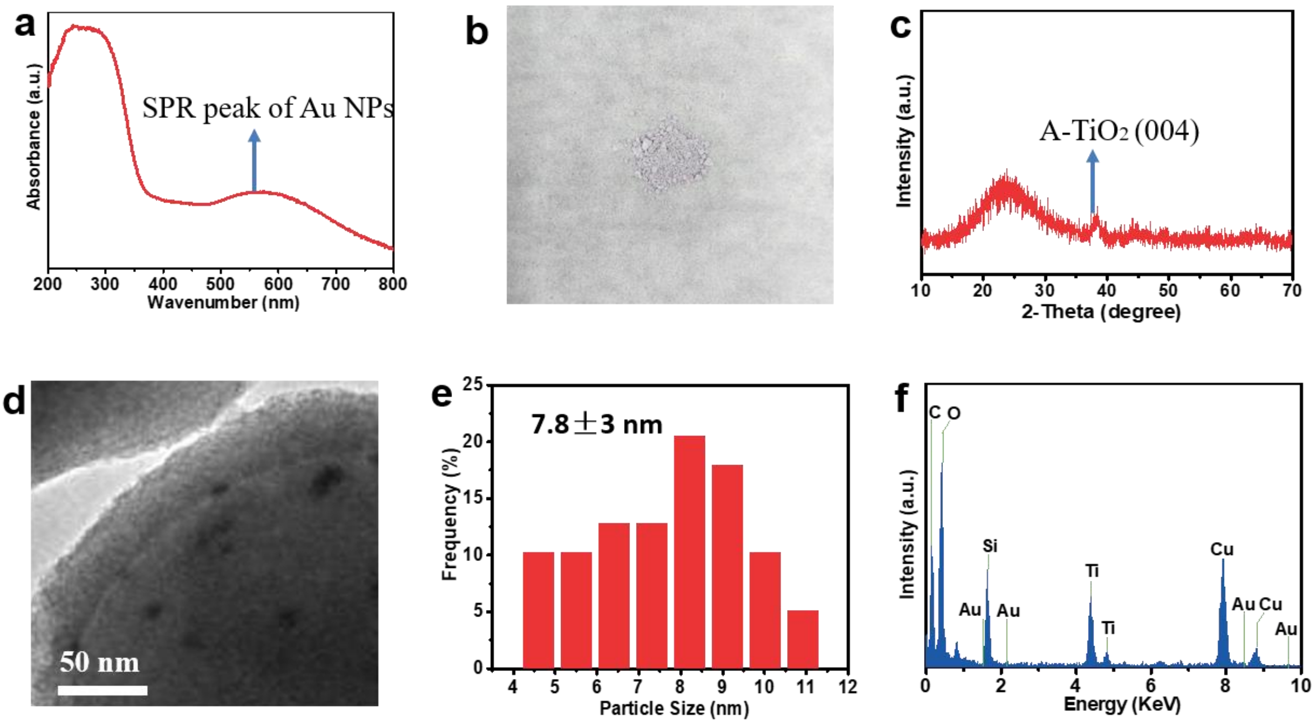

Supplementary Figure 24. Characterizations of SABT-0.15-350 composites (a) DRS spectrum; (b) digital photograph; (c) XRD pattern; (d) TEM image of SABT-0.15 composite calcinated at 350 ℃ under Ar for 2 h (denoted as SABT-0.15-350); (e) size distribution histogram of Au nanoparticles over SABT-0.15-350 sample; (f) EDX spectrum of SABT-0.15-350 originated from Supplementary Fig. 24d.

Note: The sample of SABT-0.15 has been calcinated at 350 ℃ under Ar for 2 h and the calcinated sample is denoted as SABT-0.15-350. Even though the peak of anatase TiO_2 (A-TiO_2) can be observed in the XRD pattern (Supplementary Fig. 24c), the color of SABT-0.15-350 sample changes to purple, as shown in Supplementary Fig. 24b, which corresponds to the color of Au nanoparticles (NPs), indicating that the Au GSH clusters may aggregate into large Au NPs due to the high calcination temperature. Furthermore, the DRS spectrum of SABT-0.15-350 sample in Supplementary Fig. 24a exhibits a surface plasmon resonance (SPR) peak located at 550 nm belonging to the metallic Au NPs, confirming the fusion of Au GSH clusters into Au NPs with large size. More direct evidence comes from the TEM image of SABT-0.15-350 sample, as pictured in Supplementary Fig. 24d, and the size of Au NPs is demonstrated to be 7.8 nm (Supplementary Fig. 24e), which suggests that the calcination is unsuitable for enhancing the crystallinity of TiO_2 since the high temperature can lead to the aggregation of Au GSH clusters. The EDX spectrum of SABT-0.15-350 in Supplementary Fig. 24f suggests the presence of Au, Si, Ti and O elements and the detected element Cu can be attributed to the use of Cu grid, which serves as the support for TEM analysis.

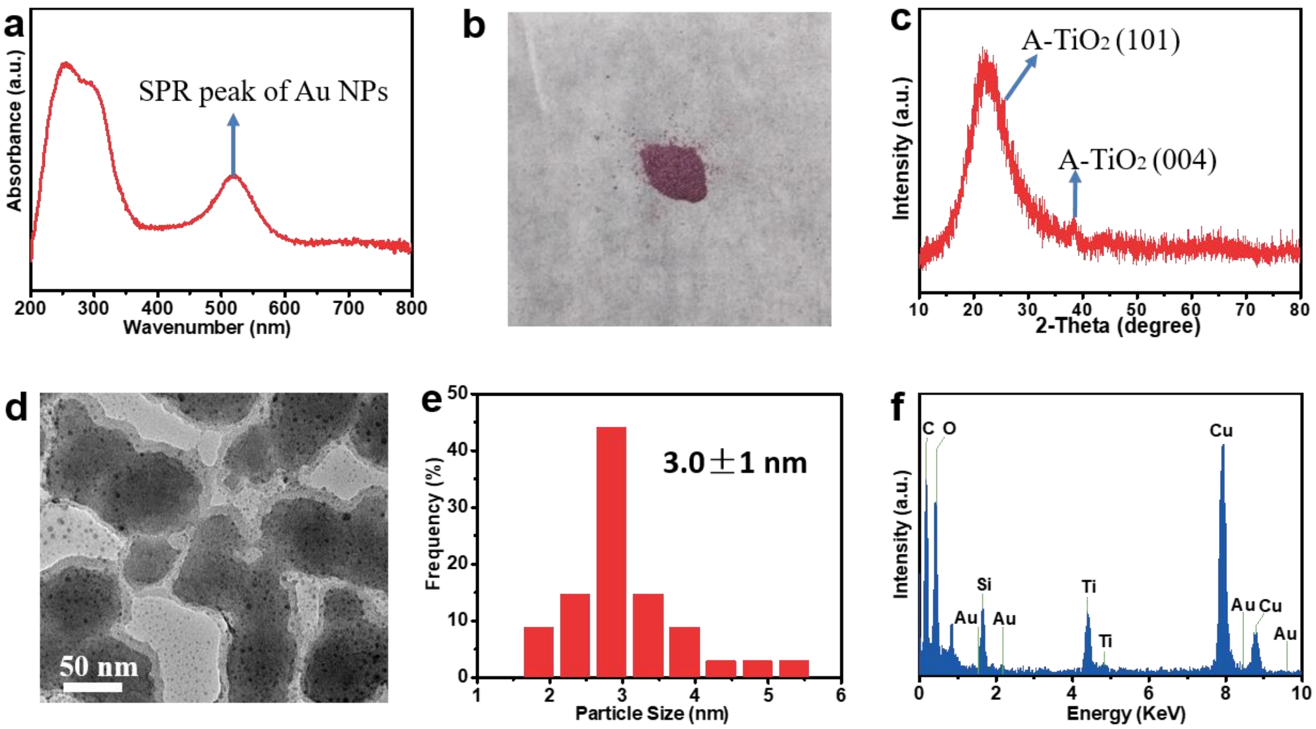

Supplementary Figure 25. Characterizations of SABT-0.15-180 composites (a) DRS spectrum; (b) digital photograph; (c) XRD pattern; (d) TEM image of SABT-0.15 composite calcinated at 180 °C for 12 h (denoted as SABT-0.15-180); (e) size distribution histogram of Au nanoparticles over SABT-0.15-180 sample; (f) EDX spectrum of SABT-0.15-180 originated from Supplementary Fig. 25d.

Note: We also treat the SABT-0.15 composite under hydrothermal condition at 180 °C for 12 h for crystallizing TiO_2 and the obtained sample is labeled as SABT-0.15-180. The application of elevated temperatures and pressures in an aqueous solution could facilitate the conversion of amorphous TiO_2 into crystalline TiO_2 and cause an increase in its crystallinity. The XRD result in Supplementary Fig. 25c indicates that the TiO_2 in SABT-0.15-180 sample is anatase (A-TiO_2). However, the intelligently designed core-shell structure of SABT-0.15 is destroyed during the hydrothermal process (Supplementary Fig. 25d). Moreover, the coalescence of Au GSH clusters to larger metallic Au NPs has also been observed and confirmed by a series of techniques, as displayed in Supplementary Fig. 25a, b and d. The size of Au NPs in SABT-0.15-180 sample is calculated to be 3.0 nm (Supplementary Fig. 25e). The EDX spectrum of SABT-0.15-180 in Supplementary Fig. 25f suggests the presence of Au, Si, Ti and O elements and the detected element Cu can be attributed to the use of Cu grid, which serves as the support for TEM analysis.

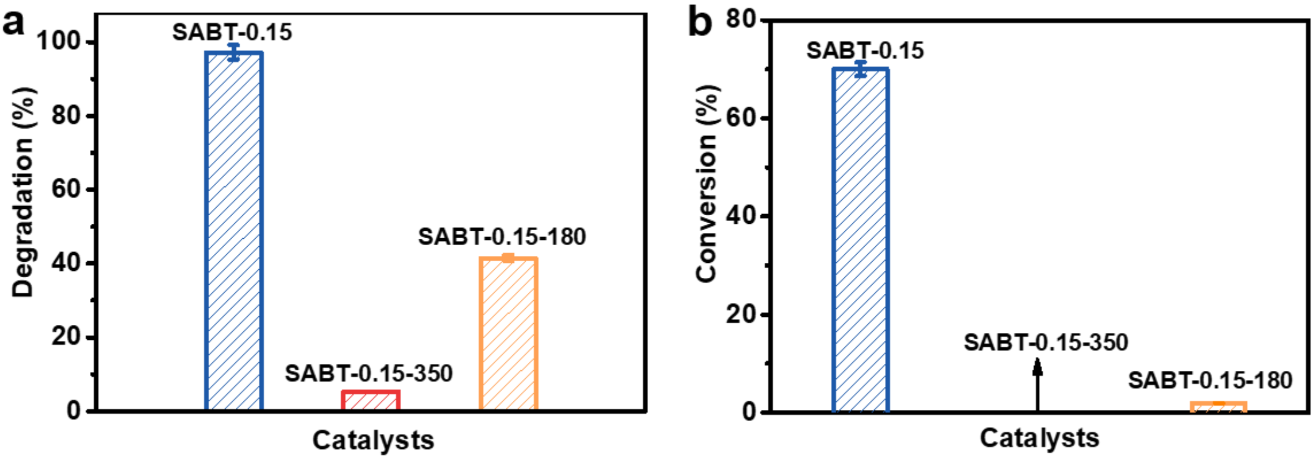

Supplementary Figure 26. Photocatalytic performances of different samples Photocatalytic degradation of (a) RhB over SABT-0.15, SABT-0.15-350 and SABT-0.15-180 composites under visible light irradiation (λ > 420 nm) for 0.5 h; photocatalytic reduction of (b) *p*-methoxy nitrobenzene to *p*-methoxy aniline over SABT-0.15, SABT-0.15-350 and SABT-0.15-180 composites under visible light irradiation (λ > 420 nm) for 5 h. Note that the error bars represent the photoactivity s.d. values calculated from triplicate experiments.

Note: The photocatalytic performances of SABT-0.15-350 and SABT-0.15-180 composites have been evaluated toward photocatalytic RhB degradation and reduction of *p*-methoxy nitrobenzene under visible light illumination (λ > 420 nm), as shown in Supplementary Fig. 26. Both SABT-0.15-350 and SABT-0.15-180 composites exhibit poor photoactivity as compared with SABT-0.15 composite, which could be arrtibuted to the aggregation of Au GSH clusters into large metallic Au NPs and the destroy of core-shell structure of SABT-0.15-180 sample.

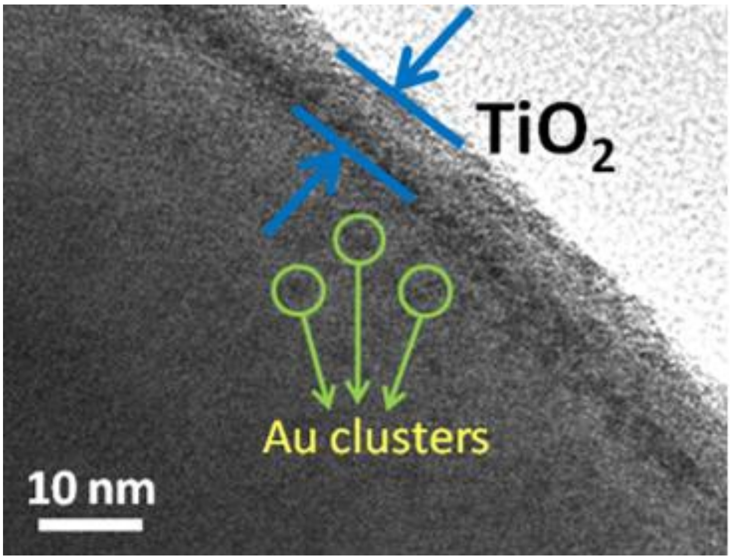

Supplementary Figure 27. Morphology information of the sample TEM image of SABT-0.05 after RhB photodegradation under visible light irradiation for 4 h.

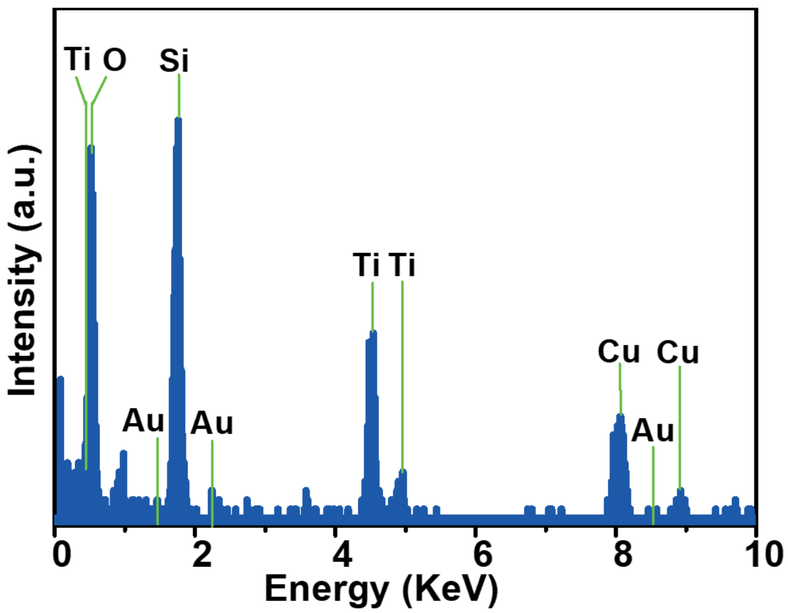

Supplementary Figure 28. **EDX spectrum** of SABT-0.05 after RhB photodegradation under visible light irradiation originated from Supplementary Fig. 27.

Note: The detected element Cu in Supplementary Fig. 28 can be attributed to the use of Cu grid, which serves as the support for TEM analysis.

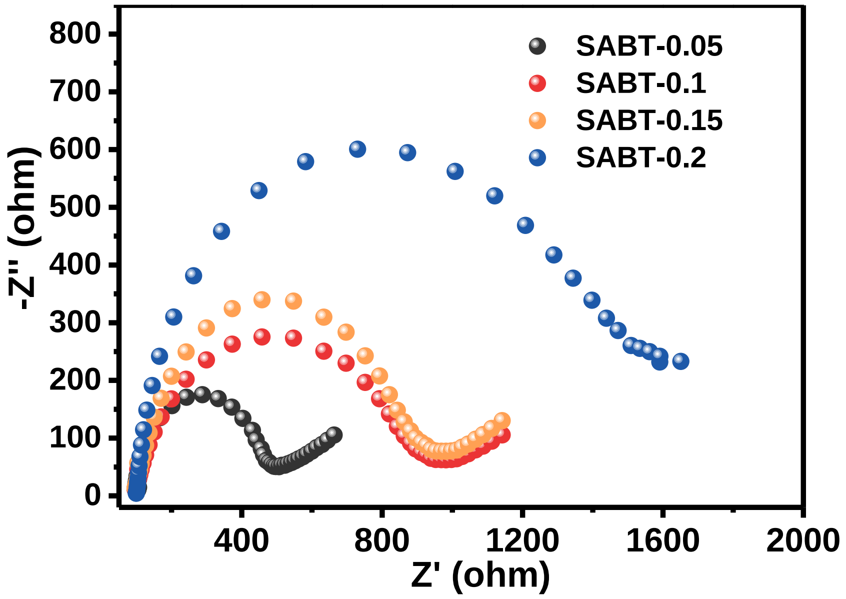

Supplementary Figure 29. Electrochemical impedance spectroscopy (EIS) Nyquist plots of SABT composites with different TiO_2 shell thickness.

Note: Electrochemical impedance spectroscopy (EIS), as a method to monitor charge transfer process on the electrode and at the contact interface between electrode and electrolyte[1-3], has been performed. It is clearly seen that the Nyquist diagrams of various SABT composites electrodes exhibit a typical semicircle at high frequency, which corresponds to the charge transfer limiting process and is ascribed to the double-layer capacitance in parallel with the charge transfer resistance at the contact interface

between electrode and electrolyte solution[1]. The SABT-0.05 electrode exhibits the most depressed semicircle at high frequency among these electrodes, suggesting that the smaller resistance and more efficient transfer of charge carriers between electrode and electrolyte solution are obtained over SABT-0.05 than those SABT samples with thick TiO_2 shell. This can be ascribed to the fact that, for the composite of SABT-0.05, the thin shell of TiO_2 layer improves the charge carrier migration of SABT composites efficiently as compared to that with thick one since the thicker layer of TiO_2 may block the transport of photogenerated electrons from Au GSH clusters to the back contact.

Supplementary Table 3. Summary of surface area and pore size of bare SiO_2 spheres, SAB and SABT composites with different TiO_2 shell thickness.

	**SiO_2**	**SAB**	**SABT-0.05**	**SABT-0.1**	**SABT-0.15**	**SABT-0.2**
**S_{BET} (m^2 g^{-1})[a]**	5.9	5.9	7.5	9.1	11.5	15.1
**Pore Size (nm)[b]**	28.6	18.6	14.5	13.7	10.2	10.1

[a] BET surface area is calculated from the linear part of the BET plot.

[b] Adsorption average pore width (4*V/A* by BET).

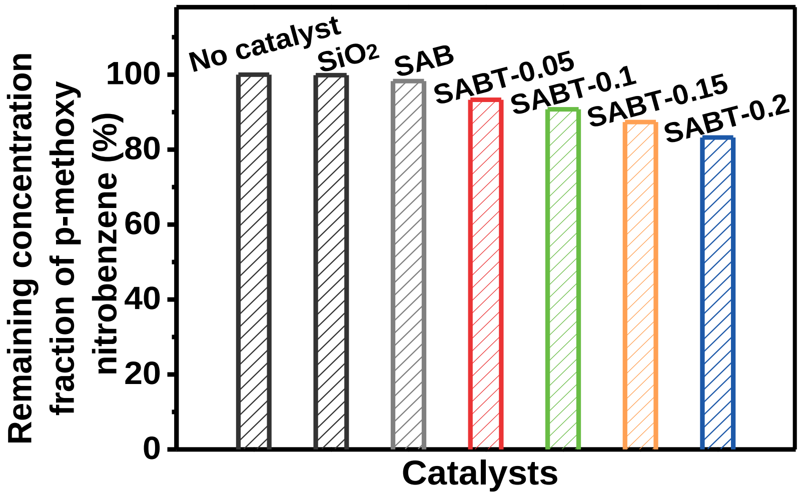

Supplementary Figure 30. Adsorption properties of different samples Bar plots showing the remaining *p*-methoxy nitrobenzene in reaction solution after being kept in dark for 3 h to achieve the adsorption–desorption equilibrium over SiO_2 spheres, SAB and SABT composites with different TiO_2 shell thickness.

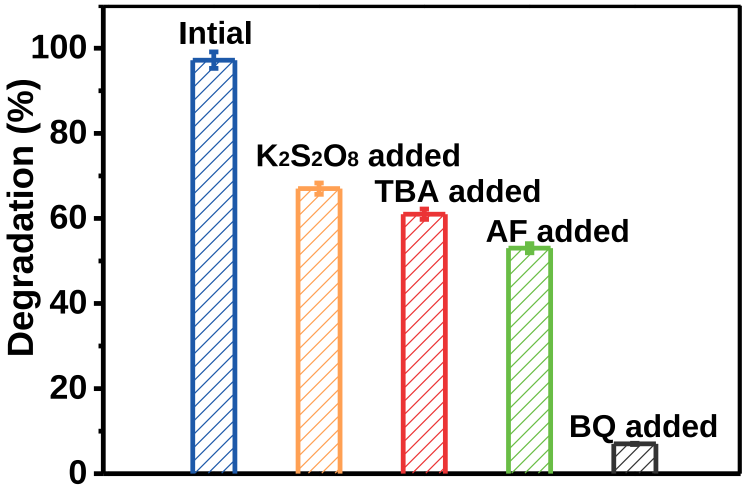

Supplementary Figure 31. Controlled experiments of photocatalytic degradation of RhB over SABT-0.15 in the presence of potassium persulfate ($K_2S_2O_8$, scavenger for electrons), tert-butyl alcohol (TBA, scavenger for hydroxyl radicals), ammonium formate (AF, scavenger for holes), *p*-benzoquinone (BQ, scavenger for superoxide radicals) under visible light irradiation (λ > 420 nm). Note that the error bars represent the photoactivity s.d. values calculated from triplicate experiments.

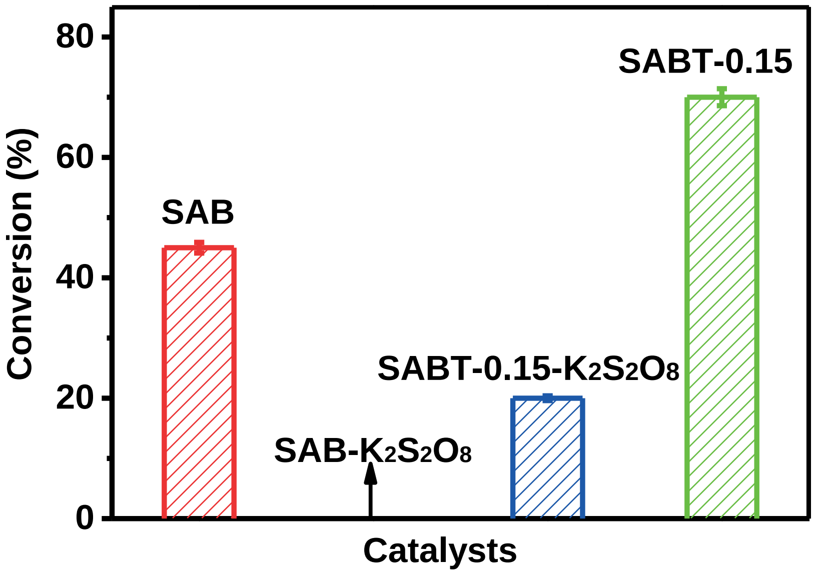

Supplementary Figure 32. Controlled experiments for photoreduction of *p*-methoxy nitrobenzene over the sample of SAB and SABT-0.15 using $K_2S_2O_8$ as scavenger for photogenerated electrons under visible light irradiation (λ > 420 nm) in an aqueous phase at room temperature. Note that the error bars represent the photoactivity s.d. values calculated from triplicate experiments.

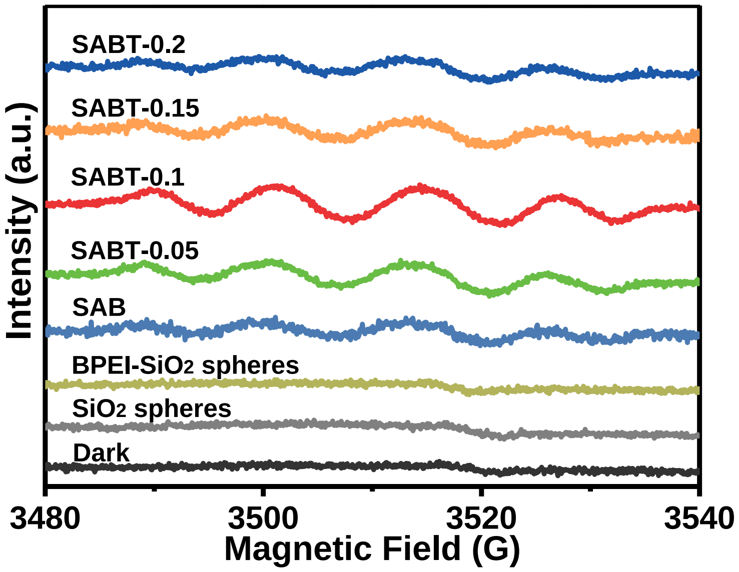

Supplementary Figure 33. Electron spin resonance (ESR) spectra of superoxide radical species trapped by 5,5-Dimethyl-1-pyrroline-N-Oxide (DMPO) over different samples under visible light irradiation (λ > 420 nm).

Supplementary Note 1.
The photostability of Au GSH clusters on the anatase TiO_2

The Au GSH clusters have been deposited on the surface of anatase TiO_2 (Supplementary Fig. 34g) *via* a pH value adjusted process and interfacial modification process for investigating the stability of Au GSH cluster on the anatase TiO_2 support. The obtained anatase TiO_2-Au GSH clusters-pH composites (ATAP) and anatase TiO_2-Au GSH clusters-BPEI composites (ATAB) have been exposed under visible light illumination (λ > 420 nm). The HRTEM image in Supplementary Fig. 34a reveals that the Au GSH clusters in ATAP suffer from fusion even after 0.5 h light irradiation, and the size of Au nanoparticles (NPs) is demonstrated to be 1.9 nm (Supplementary Fig. 34d). As for the sample of ATAB, after 3 h visible light irradiation, the size and structure of Au GSH clusters remain unchanged (Supplementary Fig. 34b and e), indicating the important role of BPEI in protecting the Au GSH clusters from being oxidized and restraining the growth of Au GSH clusters. The EDX spectrum in Supplementary Fig. 34h evidences the presence of Au, O and Ti elements over ATAB sample after light illuminated for 3 h. Unfortunately, when the irradiation time of ATAB is further extended to 5 h, the Au NPs with size of 1.8 nm are detectable, which confirms the slight aggregation of Au GSH clusters, as shown in Supplementary Fig. 34c and f.

The fusion of Au GSH clusters on the surface of anatase TiO_2 could be ascribed to the presence of abundance surface hydroxyl group [4, 5], which could facilitate formation of ·OH that decompose BPEI layer, thus resulting the formation of Au NPs with large size. The above inference has been evidenced by the synthesis of rutile TiO_2-Au GSH clusters-BPEI composites (RTAB), among which the rutile TiO_2 (Supplementary Fig. 34i) is obtained by calcinating anatase TiO_2 at 850 °C for 5 h. The sample of RTAB has been exposed to continuous visible light irradiation (λ > 420 nm) for 10 h under ambient conditions and the size information of Au GSH clusters is given by the TEM analysis. As revealed in Supplementary Fig. 12c and f , the Au GSH clusters maintain the size of 1.4 nm on the surface of rutile TiO_2 for RTAB composites after photo-irradiation. In constrast, the sample of rutile TiO_2-Au GSH clusters-pH (RTAP) has also been fabricated and irradiated under visible light for 10 h, as illustrated in Supplementary Fig. 35. The Au GSH clusters have aggregated into Au NPs with size of 3 nm, which indicates the effect of BPEI modification on enhancing the stability of Au GSH clusters under visible light illumination.

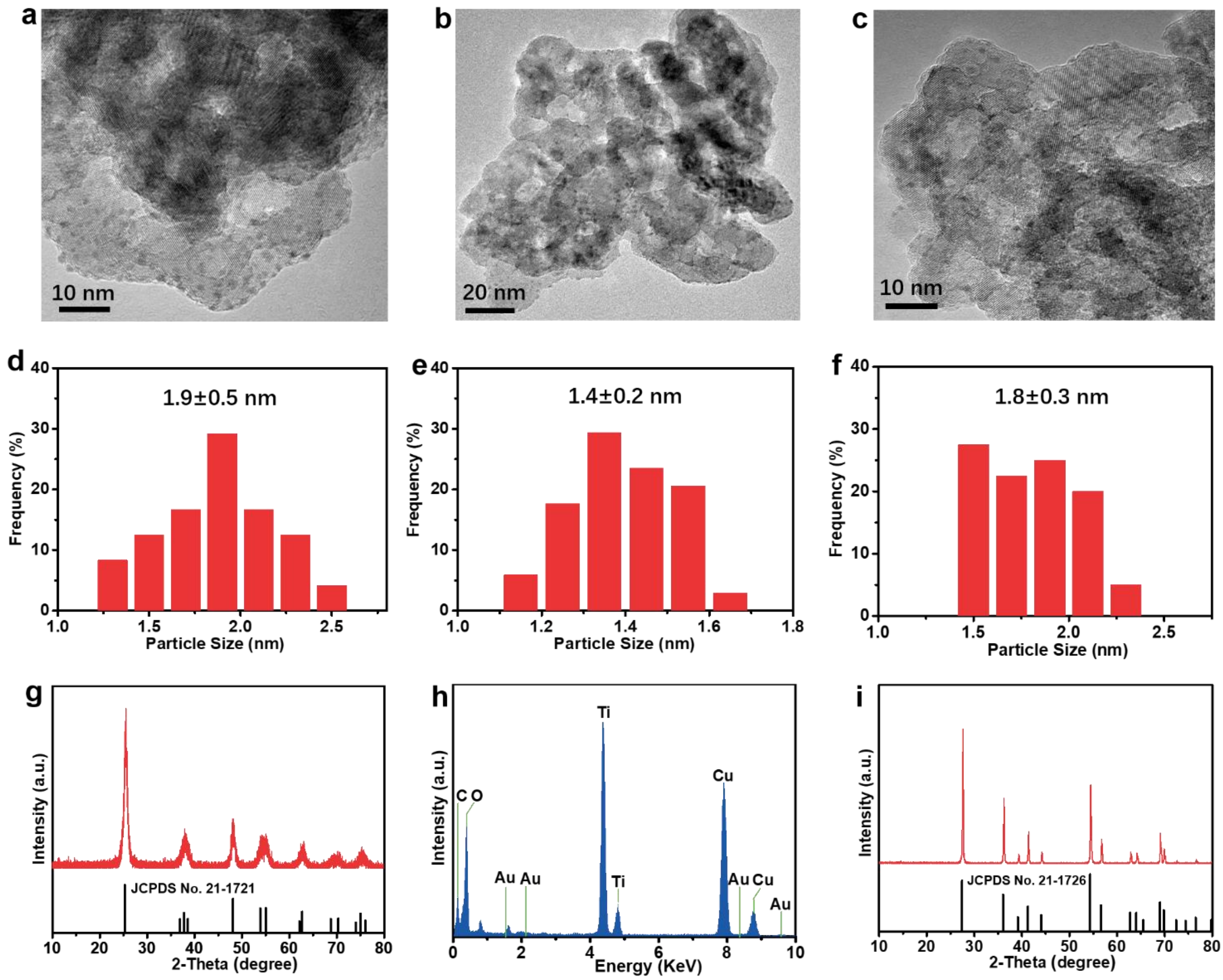

Supplementary Figure 34. Characterizations of different samples HRTEM images of (a) anatase TiO_2-Au GSH clusters-pH composites (ATAP) after visible light irradiation (λ > 420 nm) for 0.5 h; HRTEM images of anatase TiO_2-Au GSH clusters-BPEI composites (ATAB) after visible light irradiation (λ > 420 nm) for (b) 3 h and (c) 5 h; size distribution histogram of Au GSH clusters over (d) ATAP after visible light irradiation (λ > 420 nm) for 0.5 h; size distribution histograms of Au GSH clusters over ATAB after visible light irradiation (λ > 420 nm) for (e) 3 h and (f) 5 h; XRD pattern of (g) anatase TiO_2; (h) EDX spectrum of ATAB after visible light irradiation (λ > 420 nm) for 3 h; XRD pattern of (i) rutile TiO_2.

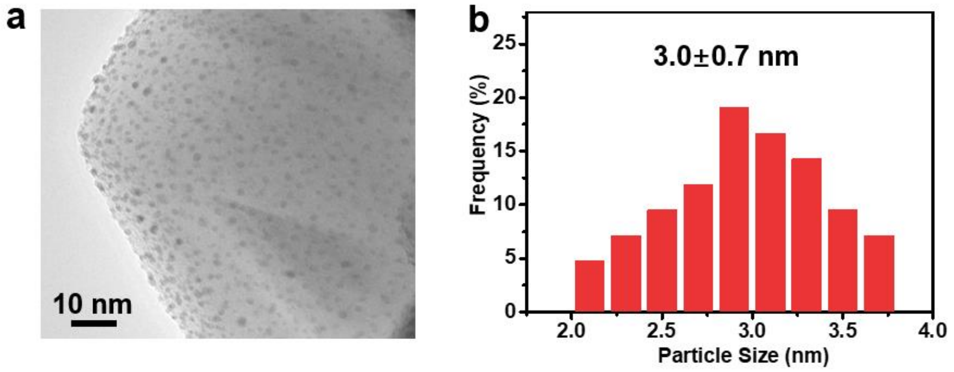

Supplementary Figure 35. Morphology and size information of Au GSH clusters in rutile TiO_2-Au GSH clusters-pH composites (RTAP) HRTEM image of (a) RTAP and size distribution histograms of Au GSH clusters over (b) RTAP after visible light irradiation (λ > 420 nm) for 10 h.

Supplementary References

1. Qiu B, Xing M, Zhang J. Mesoporous TiO_2 Nanocrystals Grown in Situ on Graphene Aerogels for High Photocatalysis and Lithium-Ion Batteries. *J. Am. Chem. Soc.* **136**, 5852-5855 (2014).
2. Weng B, Quan Q, Xu Y-J. Decorating geometry- and size-controlled sub-20 nm Pd nanocubes onto 2D TiO_2 nanosheets for simultaneous H_2 evolution and 1,1-diethoxyethane production. *J. Mater. Chem. A* **4**, 18366-18377 (2016).
3. Weng B, Xu Y-J. What if the Electrical Conductivity of Graphene Is Significantly Deteriorated for the Graphene–Semiconductor Composite-Based Photocatalysis? *ACS Appl. Mater. Interfaces* **7**, 27948-27958 (2015).
4. Liu F, *et al.* Transfer Channel of Photoinduced Holes on a TiO_2 Surface As Revealed by Solid-State Nuclear Magnetic Resonance and Electron Spin Resonance Spectroscopy. *J. Am. Chem. Soc.* **139**, 10020-10028 (2017).
5. Nie L, Yu J, Li X, Cheng B, Liu G, Jaroniec M. Enhanced Performance of NaOH-Modified Pt/TiO_2 toward Room Temperature Selective Oxidation of Formaldehyde. *Environ. Sci. Technol.* **47**, 2777-2783 (2013).
